# GPCR activation mechanisms across classes and macro/microscales

**DOI:** 10.1038/s41594-021-00674-7

**Published:** 2021-11-10

**Authors:** Alexander S. Hauser, Albert J. Kooistra, Christian Munk, Franziska M. Heydenreich, Dmitry B. Veprintsev, Michel Bouvier, M. Madan Babu, David E. Gloriam

**Affiliations:** 1 Department of Drug Design and Pharmacology, University of Copenhagen, Copenhagen, Denmark; 2 Department of Molecular and Cellular Physiology, Stanford University School of Medicine, Stanford, CA USA; 3 Department of Biochemistry and Molecular Medicine, Institute for Research in Immunology and Cancer, Université de Montréal, Montreal, Quebec Canada; 4 Laboratory of Molecular Biology, Cambridge Biomedical Campus, Cambridge, UK; 5 Centre of Membrane Proteins and Receptors (COMPARE), University of Nottingham, Nottingham, UK; 6 Division of Physiology, Pharmacology & Neuroscience, School of Life Sciences, University of Nottingham, Nottingham, UK; 7 Department of Structural Biology and Center for Data Driven Discovery, St. Jude Children’s Research Hospital, Memphis, TN USA; 8 Present Address: Data Tools Department, Novozymes A/S, Copenhagen, Denmark

**Keywords:** Structural biology, Computational biology and bioinformatics, Molecular biology, Drug discovery

## Abstract

Two-thirds of human hormones and one-third of clinical drugs activate ~350 G-protein-coupled receptors (GPCR) belonging to four classes: A, B1, C and F. Whereas a model of activation has been described for class A, very little is known about the activation of the other classes, which differ by being activated by endogenous ligands bound mainly or entirely extracellularly. Here we show that, although they use the same structural scaffold and share several ‘helix macroswitches’, the GPCR classes differ in their ‘residue microswitch’ positions and contacts. We present molecular mechanistic maps of activation for each GPCR class and methods for contact analysis applicable for any functional determinants. This provides a superfamily residue-level rationale for conformational selection and allosteric communication by ligands and G proteins, laying the foundation for receptor-function studies and drugs with the desired modality.

## Abstract (web summary)

Comparative analysis of inactive/active-state structures reveals molecular mechanistic maps of activation of the major GPCR classes. The findings and new approaches lay the foundation for targeted receptor-function studies and drugs with desired modalities.

## Main

GPCRs are nature’s primary transmembrane transducers for carrying signals from extracellular ligands to intracellular effectors, to regulate numerous physiological processes. The most widely used nomenclature designates GPCR classes A–F and was introduced in the first version of the GPCR database, GPCRdb^1^, on the basis of conserved sequence fingerprints^2,3^. The human GPCRs have also been classified on the basis of phylogenetic analysis into families (classes): glutamate (C), rhodopsin (A), adhesion (B2), Frizzled (F), secretin (B1)^4^ and Taste 2 (T, reclassified as a separate family in ref. ^5^). The human GPCRs are activated from different endogenous ligand-binding sites in the transmembrane (class A) or extracellular domain (class C) or both (classes B and F), and have very low sequence similarity (cross-class pairs, mean 23%). This raises the question as to what extent the GPCR superfamily utilizes universal or unique activation mechanisms. Considering that GPCRs mediate the actions of two-thirds of endogenous hormones and neurotransmitters^6^, and over one-third of drugs^7–9^, mapping their activation mechanisms is important to understand human physiology, disease etiology and for rational drug design.

Comparisons of inactive and active structures of class A GPCRs have uncovered common activation mechanisms within the seven transmembrane helices (7TM), which have been shown to tilt, rotate, elongate or switch residue side chain rotamers to create contact networks that stabilize the receptors in a specific state^10–13^. These contacts converge near the G-protein site^14^ and are conserved regardless of the subtypes of intracellular effector^11^. Site-directed mutagenesis of contact residues has revealed differences in pharmacological responses, including constitutive activity^11^ and signaling bias^15^, and naturally occurring mutations have been associated with disease or change of response^11,16,17^. However, the classes B, C and F are largely unexplored with respect to common activation mechanisms, and until now such studies have not been possible due to a lack of structures across the activation states.

**Fig. 1:**
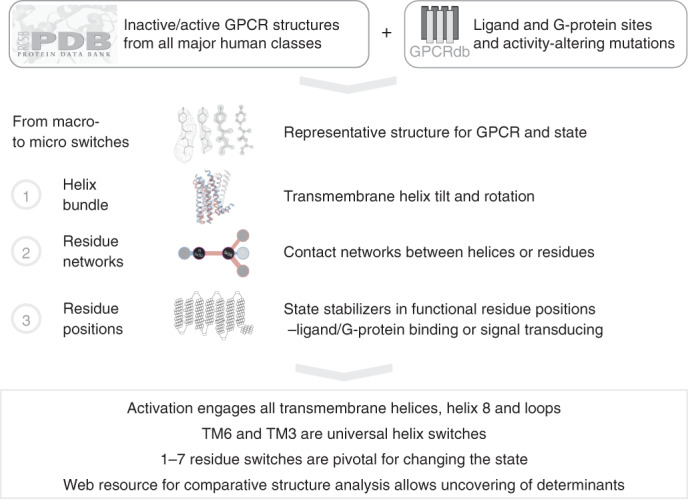
Analysis pipeline for elucidation of GPCR activation mechanisms. Pipeline for analysis of universal and distinct activation of macro/microswitches spanning helix repacking to side chain rotation and the connection to ligand-binding, G-protein coupling and signal transduction sites (Methods).

In this work, we conducted a comprehensive comparative structural analysis of inactive/active-state structures in each GPCR class by analyzing all 488 available structures from different GPCR classes (Fig. 1). We present a GPCR superfamily-wide molecular mechanistic map of activation, and link determinants to ligand-binding, G-protein coupling, transduction and allosteric sites. This can serve as a roadmap to assess the activation of any GPCR and to understand how common or distinct determinants can fine-tune physiological signaling responses and contribute to a desired drug efficacy.

## Results

### Dataset and methods for comparative structure analysis

To present the best maps possible to date, we made a comprehensive annotation of all available class A, B1, C and F GPCR structures (the 406 class A GPCR structures are twice as many as in any previous report^11^). Stringent quality filters were applied to these structures to select the representative inactive/active-state structures from each class (Methods and Supplementary Data 1). Out of 68 templates used, 45 have a resolution of 3.0 Å or better. It should be noted that in lower resolution structures (for example, above 3.0 Å) it is mainly backbone movements that can be discerned, while residue contacts can only be under-represented (that is, not lead to false-positive contacts). For classes A and B1, 22 out of 58 and 5 out of 5 receptor families (based on a shared endogenous ligand), respectively, have a structural template. This diversity ensures that any artifacts observed in a specific position or structure will only have a marginal effect on the overall frequency of movements and contacts.

We introduce a definition of state-specific contacts on the basis of their relative frequency (%) in a given inactive/active-state structure. Similarly to the scores calculated in ref. ^11^, this allows for the identification of many more determinants than requiring 100% presence and absence, respectively, in opposite states. An example is the four inactive- and two active-state stabilizing contacts identified in the landmark study comparing five class A GPCRs across the states^14^ (in which only two and three active-state templates were G protein- and agonist-bound, respectively). Importantly, the use of frequencies with (different) sets of inactive- and active-state structures opens up not only the same, but also different receptors across the states, for analysis, since the same helix backbone movements and contacts can be mediated by different amino acids. This is critical to the identification of a comparable number of residues with state-specific contacts (‘state-determinant residues’) in classes C and F, for which the structural coverage is limited so far: five and three receptors fulfill our cut-offs, representing 23% and 27% of all members of these relatively small classes, respectively. Furthermore, we also apply residue-pair conservation cut-offs (Methods). Together, the combined contact frequencies and conservation cut-offs address the class representativeness, allowing analyses across the GPCR superfamily.

### GPCR activation engages all TMs with unique contact patterns

**Fig. 2:**
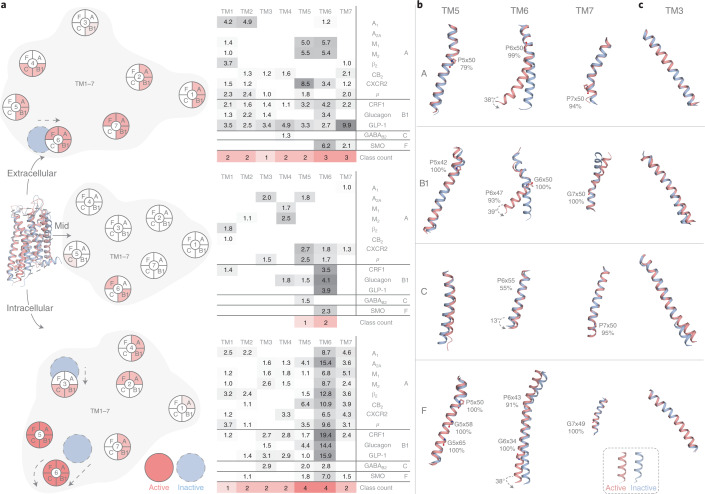
Transmembrane helix movement upon activation, and universal TM3 and TM6 helix ‘macroswitches’. **a**, Movements (Å) over 1.0 Å at the extracellular end, membrane mid (determined using ref. ^43^) and intracellular end of the transmembrane helices TM1–7 upon comparison of all available receptor inactive- and active-state structure pairs (Supplementary Table 1). Red intensity denotes the number of classes with a consensus. **b**, Movement and conserved hinges of TM6 and the adjacent TM5 and TM7. **c**, TM3 cytosolic tilt and overall rotation. **b**,**c**, GPCR class-representative inactive/active receptor structure pairs: A: β_2_ (refs. ^44,45^), B1: GLP-1 (refs. ^18,22^), C: GABA_B2_^46^ and F: Smoothened^47,48^ receptors. Proline and glycine residues that increase helix plasticity are shown, along with their percentage conservation in the GPCR class.

**Extended Data Fig. 1:**
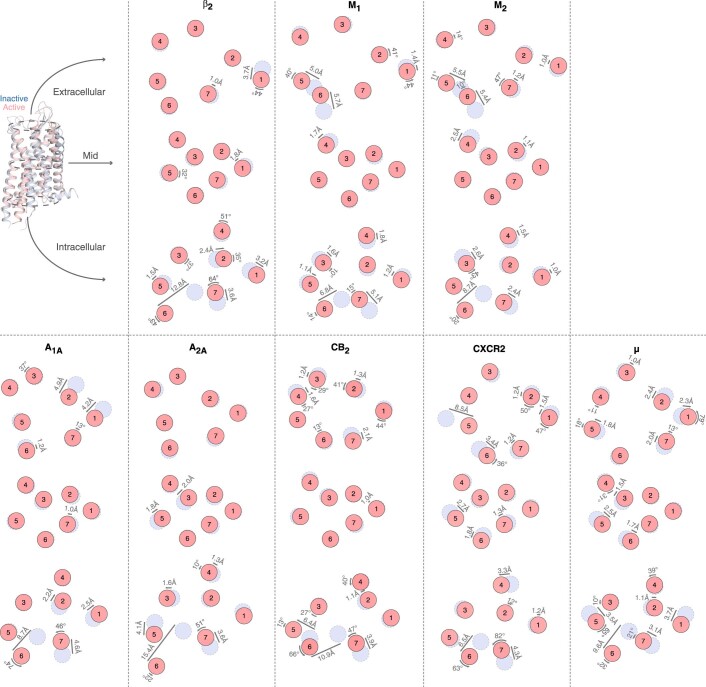
TM1-7 movement of class A receptor pairs upon activation. Transmembrane helix movement at the extracellular face, membrane mid and intracellular face based on comparison of representative inactive and active state structure pairs of class A GPCRs (Extended Data Table 1).

**Extended Data Fig. 2:**
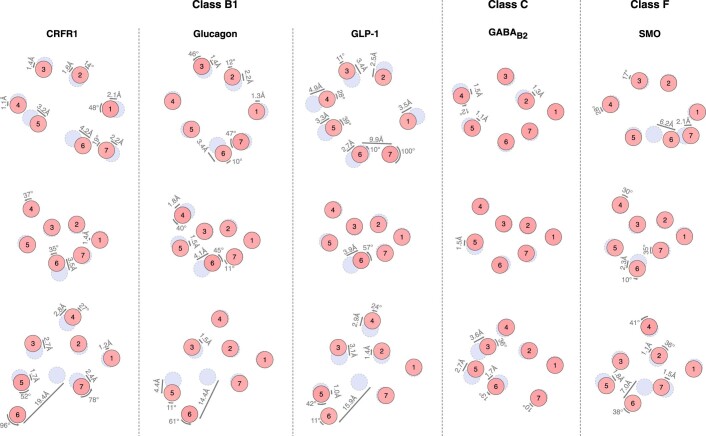
TM1-7 movement of class B1, C and F receptor pairs upon activation. Transmembrane helix movement at the extracellular face, membrane mid and intracellular face based on comparison of representative inactive and active state structure pairs of class B1, C and F GPCRs (Extended Data Table 1).

To investigate how the seven transmembrane helices TM1–7 rearrange upon activation, we compared the 13 GPCRs for which both an inactive- and active-state structure are available (all active templates are in the G-protein-bound state, Supplementary Table 1). We find that all transmembrane helices rearrange at least one end by >1.0 Å in most receptors, thus demonstrating unappreciated structural dynamics (Fig. 2, with individual receptor plots in Extended Data Figs. 1 and 2). Notably, in class B1, all seven transmembrane helices relocate their extracellular ends where the N-terminal domain restricts the conformation of the 7TM before activation^18^. Glucagon-like peptide 1 (GLP-1), which is the best template having a full-length inactive structure^18^, has a 2.5–10-Å relocation of TM1–7 in this region; all of which also move in corticotropin releasing factor type 1 (CRF1). Furthermore, we analyzed the conformational change of each class at the membrane mid, extracellular end and cytosolic end. Here the rearrangements total 6, 12 and 28 Å, respectively, and involve on average 1.7, 4.4 and 4.5 transmembrane helices per receptor. This presents a quantitated characterization of the magnitude and abundance of helix rearrangements at the mid, extracellular and cytosolic regions of the transmembrane domain. It supports opposite functional roles in: (1) maintaining a stable GPCR fold, (2) adapting to ligands of diverse size or (3) coupling to the much larger G proteins, respectively. This connects structure and function and reveals a wide engagement of the GPCR fold across its helices and domains.

**Fig. 3:**
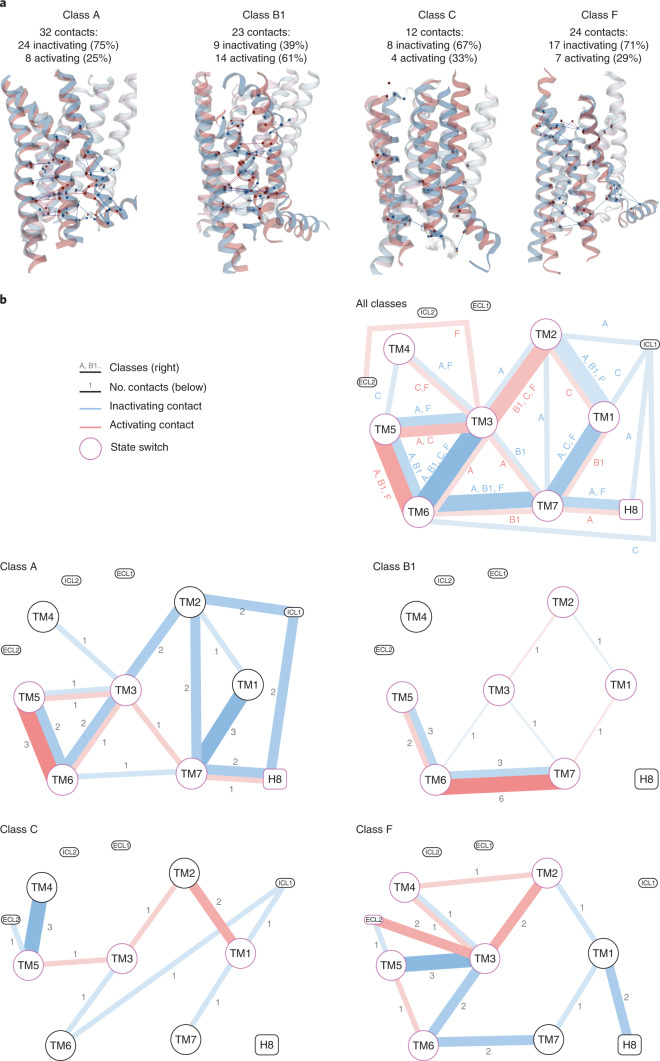
GPCRs stabilize inactive and active states by rerouting contacts between TM1–7, H8, ICL1 and ECL2. **a**, State-specific residue-residue contacts in each GPCR class visualized as lines within representative inactive/active receptor structure pairs (same as in Fig. 2b,c). Numbers indicate the total, inactivating and activating contacts in each GPCR class. **b**, Contact networks between the seven GPCR transmembrane helices, TM1–7, and the first intracellular (ICL1) and second extracellular (ECL2) loops (intrasegment contacts not shown). Line thickness represents the number of classes (top-most) or contact frequency differences between the inactive and active states. Line color indicates inactivating (blue) and activating (red) contacts. Receptor segments with a magenta border are ‘switches’, that is, having contacts across both states. Contacts are identified on the basis of a higher frequency (%) in inactive than in active-state receptors. **a**,**b**, The frequency difference threshold was set according to the structural coverage in each GPCR class (threshold: no. members, inactive/active state templates): A: 40% (285, 33/14), B1: 67% (15, 3/10), C: 75% (22, 4/2) and F: 100% (11, 2/2). To ensure that the identified determinants are applicable throughout each class, we also applied a sequence conservation cut-off requiring at least 30% of all its receptors to contain one of the amino acid pairs observed to form the given state-specific contact. Contact definitions are explained in the settings menu of the online ‘Comparative structure analysis’ tool (https://review.gpcrdb.org/structure_comparison/comparative_analysis).

**Fig. 4:**
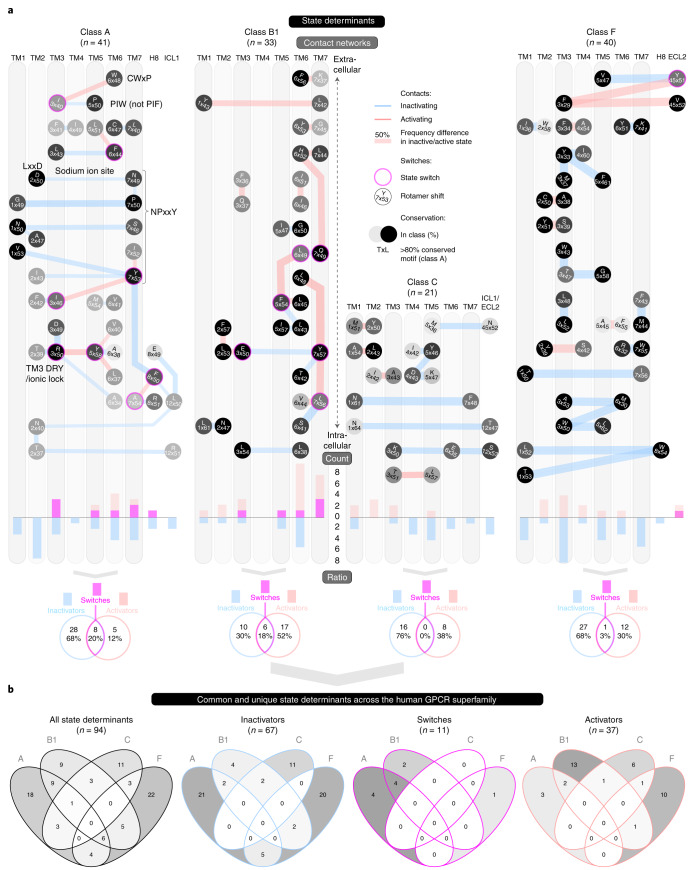
State-stabilizing contact maps and differences at the residue-level ‘microswitches’. **a**, Contact networks visualize the wiring of state determinants from the extracellular (top) to intracellular (bottom) sides. Contact frequency differences between the inactive and active states are shown as varied line thickness, and residue rotamers as rotation of the consensus amino acid in the analyzed structure and its generic residue number. Two-way Venn diagrams depict the number and percentages of inactivator (blue), activator (red) and switch (magenta) state-determinant positions. Bar diagrams show their distribution across the TM helices, H8 and loops. **b**, Comparison of common and unique state-determinant positions across all investigated classes.

We next investigated how the rearrangements of the transmembrane helix bundle change the residue contact networks between receptor segments by comparing all 42 inactive-state and 27 active-state representative structures (all active templates are G-protein bound, Supplementary Table 1). We find that, in addition to TM1–7, state-specific contacts are also formed to helix 8 (H8) in classes A and F, intracellular loop 1 (ICL1) in classes A and C and extracellular loop 2 (ECL2) in classes C and F (Figs. 3 and 4). Classes A, C and F have two-thirds to three-quarters of inactivating contacts (75%, 67% and 71%, respectively). Class B1 has fewer (39%) inactivating contacts, but this figure is close to half (namely, 48%) when considering only the contacts spanning different segments (excluding four intrahelical contacts). Sequence conservation analysis of each GPCR class shows that most residue-residue contacts can be formed in at least 30% of receptors. These findings provide a structural rationale of why most receptors have no or little activity without prior stimulation by an agonist. Furthermore, it demonstrates that most TM helices can switch from mainly inactive- to active-state contacts (Fig. 3b). Notably, this rewiring leads to markedly different patterns of segment contacts across GPCR classes, which combine several unique and some common contacts (below), as even high-homology receptors sharing endogenous ligands, such as the A_1_ and A_2A_ adenosine or muscarinic acetylcholine M_1_ and M_2_ receptors, display unique helix movements (Fig. 2).

The common movements and contacts (see below) provide a structural rationale for shared overall functions throughout the GPCR superfamily: for example, ligand-dependent activation, signal transduction across the cell membrane and G-protein coupling. These are complemented by a larger number of unique structural features that allow the receptors to be diversified with respect to the specific ligand scaffold, G-protein profile and functional response kinetics and efficacy.

### TM6 universal helix ‘macroswitch’ differs mechanistically

We next investigated single helix rearrangements across 13 receptor inactive/active-state structure pairs. We find that outward movement and rotation of TM6 on the cytosolic side—opening for G-protein coupling—is a universal feature of activation throughout the GPCR superfamily (Fig. 2a), as first suggested for class A^19^. Intracellular TM6 movement is observed in all 13 receptors investigated and is largest in classes A (7–13 Å), B1 (14–19 Å) and F (7 Å), where it is combined with a substantial average rotation: 38, 39 and 38°, respectively. The extracellular end of TM6 in these classes also moves by on average 2.0, 3.4 and 6.2 Å, respectively. The movement of TM6 gives it the largest (B1 and F) or second largest (A) number of contacts stabilizing an inactive or active state in these classes (Fig. 3); however, TM6 also displays large mechanistic differences across classes. Class B1 has a unique unwinding of the extracellular-facing half of TM6 in all three activated receptors^20–22^. This corroborates a recent study comparing the class B1 glucagon and class A β_2_ receptors, linking the weaker ability of the agonist to induce the outward movement of cytoplasmic TM6 to a slower G-protein activation^21^. Furthermore, in class C, TM6 uniquely has no extracellular movement and only a small cytosolic movement (2.8 Å) and rotation (13°). Consequently, TM6 has only two state-specific contacts and stabilizes only the inactive state, while TM1, TM3 and TM5 have 3–5 contacts across both states (Fig. 3b). The smaller role of TM6 in activation throughout the class is supported by markedly lower conservation of its proline, 55% compared to 93–100% in the other classes A–C, while the importance of TM7 is emphasized by a 95% conserved proline (Fig. 2b). The noncanonical small TM6 tilt, with a less conserved kink and atypical contacts to the adjacent TM5 and TM7, is associated with a different overall mechanism. This is because class C GPCRs work as an asymmetric homo- or heterodimer and the activated transmembrane domain is mainly characterized by dimer reorientation rather than rearrangements within the receptor monomers, of which only one couples to a G protein.

These findings show that the GPCR activation machinery utilizes TM6 as a universal switch in each class, although undergoing different rearrangements, such as helix toggling, rotation and/or unwinding. Such commonality across classes in the activation mechanism at the level of transmembrane helices, but diversity and nuances of the type of movements, provides an important structural rationale for drug discovery and in the future design of experiments to elucidate the effects of mutations, ligand efficacy and G-protein selectivity^23^ mechanisms.

### TM5 is a common switch and TM3 is a hub for stabilization

TM5, like TM6, can be considered a universal switch for GPCR activation, as it moves on the intracellular side in all four classes (with, on average, A: 2.1, B1: 2.4, C: 2.0 and F: 1.8 Å) (Fig. 2b and Extended Data Fig. 2). TM7 also moves in all classes except class C, either on the cytosolic side (all class A receptors), the extracellular side (for example, 10-Å movement and 100° rotation in GLP-1, the class B1 receptor with full-length templates) or on both sides (class F). These movements are possible due to several conserved proline and glycine residues that induce helix plasticity (Fig. 2b). Class A GPCRs have triple proline kinks in TM5–7, which allow TM5 and TM7 to close in on and stabilize TM6. Class B1 TM6 unwinding is facilitated by both a proline kink (P6×47) and a glycine (G6×50). Class B1 GPCRs also feature a proline kink (P5×42) near the extracellular end of TM5, which moves 3.3 Å in GLP-1, and a glycine kink (G7×50) in TM7. Class F combines the TM6 switch with movements of cytosolic TM5 (with one Pro and two Gly residues) and TM7 on both sides. This demonstrates that TM6 does not act on its own but is supported by TM5 and TM7 in a concerted movement and that the determinants of this plasticity are conserved throughout the classes.

We find that TM3 has the largest number of state-specific contacts to other receptor segments in each GPCR class (3–5, Fig. 3). This reveals that TM3, previously shown to be a stabilization hub maintaining the common transmembrane fold^24^, also plays a central role in stabilization of distinct states across the GPCR superfamily. Furthermore, whereas an early report based on two class A GPCRs suggested an activation mechanism involving an upward movement of TM3 (ref. ^25^), our analysis of TM helix movements across classes instead points to rotation as the main mechanism (Extended Data Fig. 1). In 11 out of 13 of the investigated receptor pairs (all except A_2A_ and CRF1), TM3 rotates at either the cytosolic (most frequent for class A, average 16°) or extracellular end (classes B1, C and F, average 19°, 12° and 17°, respectively), while no receptor rotates at both ends or at the membrane mid (Extended Data Figs. 1 and 2). Lateral movement of TM3 contributes to a different extent across classes, being mainly at: both ends (B1), the cytosolic end (C), either end (a minority of receptors in A) or no movement (F). In contrast, TM4, which is peripherally located in the transmembrane helix bundle, has few or no contacts (class A:1, B1:0, C:3 and F:0). This shows that the abundant helix packing has not immobilized TM3. Instead, it contributes to GPCR activation in several places through an array of mainly local rotations or movements.

### Residue ‘microswitches’ expand the class A activation model

To investigate state determinants at the residue ‘microswitch’ level, we indexed topologically corresponding receptor positions with generic residue numbers^26^ and classified them into ‘inactivators’, ‘activators’ and ‘switches’ on the basis of frequent contacts in inactive, active and both states, respectively. We uncovered a comparable number of state determinants across classes (Fig. 4a). Across the classes, these span 94 distinct residue positions, 67 inactivators, 37 activators and only 11 switches (Fig. 4b and Supplementary Table 2). Only nine switches undergo side chain rotamer shifts, revealing that the rotamer microswitches—described as major state determinants for class A GPCRs^12,13^—play a small role in the GPCR superfamily. Importantly, many determinant positions contain the same highly conserved amino acid (Fig. 4a), and the amino acid pairs observed for each contact are conserved in at least 40% of all receptors. Notably, this includes the reference positions for generic residue numbers (index ×50) in five out of seven TMs, H8 and ICL1, all major previously known class A state determinants^11,12^ and a class F switch, 6×32 (refs. ^27,28^) (sequence motifs and microswitches with magenta border in Fig. 4a). This demonstrates that our approach (Discussion) identifies both known and new conserved determinants, even where the structural coverage is limited, including the active state of classes C and F.

In class A, W6×48, earlier suggested to be a rotamer toggle switch^12^, does not itself undergo a rotamer shift but a helix rotational shift (of 10°) and is approached by I3×40, which has both types of rotation. This provides an alternative to a reported ‘PIF’ motif^29–31^, which is here found to consist most frequently of ‘PIW’ (P5×50 being the third residue). The PIF motif’s last residue, F6×44, rather acts as a switch in a new triplet ‘LLF’ located in the same helices: TM3, TM5 and TM6. The two residues D2×50 and N7×49, which coordinate a sodium ion^32^, are here found to have a direct interaction that stabilizes the inactive state. Notably, N7×49 contacts P7×50, uncovering a concerted stabilization across the sodium ion site and TM7 helix kink around the ‘NPxxY’ motif. The final residue of the NPxxY motif, a Y7×53 switch^11–13^, has three inactivating and two activating state-specific contacts with over 40% frequency difference, including to another switch: I3×46. The TM3 ‘DRY’ motif includes an intrahelical ionic lock from D3×49 to R3×50, which, upon activation, swings to interact with the G protein as well as the switch Y5×58 on TM5 (ref. ^13^). Of note, the restraining of R3×50 is strengthened by contacts to TM2 and TM6 (A6×34)—however, typically not to E6×30, which was part of the first ionic lock reported in rhodopsin^33^ but less conserved (E: 25% or D/E: 31% compared to D: 65% or D/E: 86% for position 4×49). On the intracellular side, H8 and ICL1 contain three and two determinants, respectively, including the novel switch F8×50 contacting another new switch on TM7, A7×54. These findings corroborate the findings of previous studies on class A^10–14^. They also, together with the concerted movement of TM5–7 and the role of TM3 as a state-stabilization hub (see above), substantially expand the activation model of class A receptors.

**Fig. 5:**
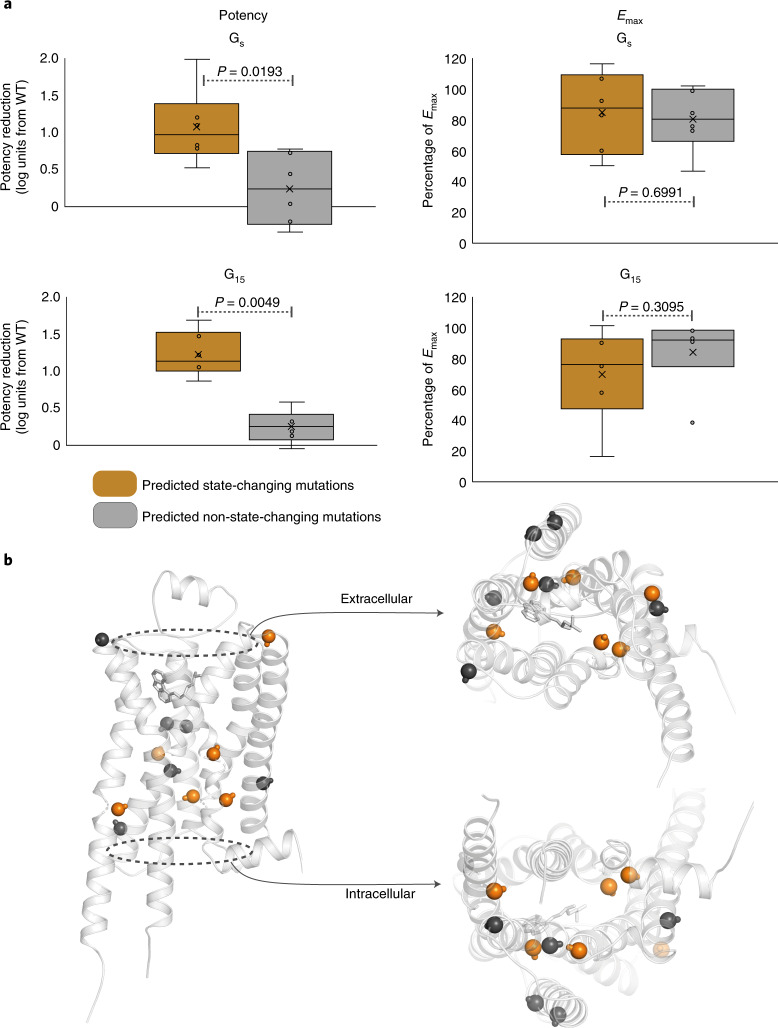
Mutations of predicted state-changing residue positions reduce potency. **a**, Effect upon alanine mutation of predicted state-changing and nonstate-changing residues, respectively, on epinephrine-induced β_2_-adrenoceptor activation of G_s_ and G_15_ measured by BRET-based biosensors. Predicted state-changing residues show a significantly higher reduction in potency (left), but not in efficacy (*E*, right), for both G_s_ and G_15_ relative to wild type. Statistical significance has been assessed by a two-sided Wilcoxon rank-sum test (*n* = 6 for each category, individual data points in Supplementary Table 2). Box-and-whiskers plots are presented with interquartile box bounds (25% and 75%); middle line represents the median; x represents the mean; whiskers extend to the minimum and maximum value. **b**, Structural mapping of predicted state-changing (orange) and nonstate-changing mutations (gray) on the inactive carazolol-bound inactive β_2_-adrenoceptor structure (PDB 2RH1)^44^. Cα are shown as spheres and Cα-Cβ bonds are displayed as sticks. Five out of six state-changing mutations cluster tightly in the transduction pathway between the ligand and G-protein pockets.

To further substantiate the importance of inactivating and activating state determinants (collectively, predicted state-changing residue positions), we performed mutagenesis experiments and measured epinephrine-induced β_2_-adrenoceptor activation of G_s_ and G_15_ using bioluminescence energy transfer (BRET)-based biosensors. We mutated six predicted state-changing (contact frequency difference >80% across states) and six nonstate-changing residues to alanine. To isolate effects due to intrareceptor conformational stabilization, these excluded residues interacting with ligands or G proteins in structure complexes. We found that state-changing positions are more prone than nonstate-changing mutations to alanine mutation-induced potency reductions for G_s_ (mean log(EC_50_) from wild type (WT) 1.07 versus 0.22 (EC_50_, half-maximum effective concentration); Wilcoxon rank-sum test: *P* = 0.0193) and G_15_ (mean log(EC_50_) from WT 1.25 versus 0.25; Wilcoxon rank-sum test: *P* = 0.0049) (Fig. 5a and Supplementary Table 2). In contrast, the mutations did not have a statistically significant differential effect on efficacy (Wilcoxon rank-sum test; G_s_: *P* = 0.6991; G_15_: *P* = 0.3095). Five out of six state-changing mutations cluster tightly in the transduction pathway between the ligand and G-protein pockets, and this group of mutations more frequently form intrahelical receptor contacts, whereas several nonstate-changing mutations instead face the membrane (Fig. 5b). This confirms the correlation between state-specific structural residue-residue contacts and ligand-induced pharmacological receptor activity, and points to reduction in potency (not efficacy) and differential intrahelical contacts as underlying determinants.

### Class B1 state-determinant residues shared with class A

**Extended Data Fig. 3:**
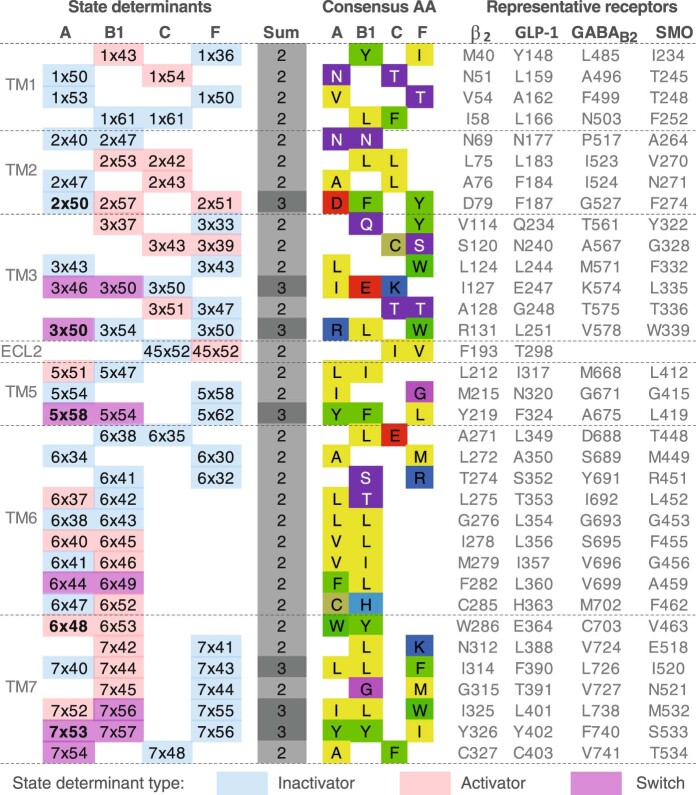
State determinants conserved across classes. Heatmap of state determinants shared by at least two GPCR classes along with their consensus amino acid and residues in representative receptors. Each row contains corresponding positions denoted with the generic residue numbers in each class^1^. Key class A state determinants are shown in bold.

The comparison of unique and common state determinants across the GPCR superfamily shows that nearly half of the B1 determinants (16 out of 33) map to equivalent topological positions as in class A, compared to 10 for class F and 8 for class C (leftmost in Fig. 4b). Furthermore, the classes B1 and A have four common switches (second to rightmost in Fig. 4b). Notably, this includes the two known microswitches (A/B1 residue number): Y5×58/F5×54 and Y7×53/Y7×57 as well as the pair I3×46/E3×50 and F6×44/L6×49 (magenta in Extended Data Fig. 3). We also find that the class A ‘toggle switch’ activator W6×48 is substituted by a conserved smaller aromatic residue Y6×53 in class B1(Extended Data Fig. 3). Together, these commonalities between classes B1 and A are indicative of partially shared activation mechanisms.

However, there are also markedly unique features in class B1. Strikingly, 14 out of 33 of the state determinants in class B1 are located in TM6, which engages 10 additional determinants (mostly in TM5 and TM7) (Fig. 4) that move together with TM6 (Fig. 2). The high concentration of state determinants in TM6, and packing to adjacent helices, may provide a plausible structural rationale for a recent report demonstrating a higher energy barrier for the formation of the kinked and partially unwound TM6 in the class B1 glucagon receptor compared to the class A β_2_-adrenoceptor^21^. Another unique feature in class B1 is two additional switches in TM7 (Q7×49 and L7×56). Class B1, like class A, has a large structural coverage (10 out of 15 receptors) and contains several major drug targets^7^. The map of state determinants in class B1 gives a better understanding of the basic receptor-activation mechanisms and presents a foundation for targeting determinant networks in structure-based design of new drugs that stabilize receptors in the desired state.

### No universal residue-level microswitch mechanism

Class C has the fewest residue state-determinant ‘microswitches’ and no activation switch with high-frequency contacts in both states. This is in concordance with its smallest (by far) conformational change (Fig. 2). Another characteristic of class C is that it has no determinants that form contacts within the same transmembrane helix, whereas each of classes A, B1 and F has four such microswitches. This reflects that class C GPCRs uniquely bind the endogenous ligand entirely in the N terminus and transduce signals across the membrane by dimers that reorient. In addition to the 7TM domain, class C GPCRs have one determinant in ECL2 and two microswitches in ICL1 (as does class A). Class F has 40 residue microswitches, of which one is in H8 (8×54) and two in ECL2 (Y45×51 and V45×52). The two ECL2 positions follow a conserved cysteine C45×50, which forms a covalent disulfide bridge, also to TM3, across the GPCR classes^34^. Class F has the highest conservation of determinant consensus amino acids (on average 82%) showing that most contacts identified in the three structural templates: FZD4, FZD7 and Smoothened, are probably shared by the remaining eight receptors. Class F has one switch, Y45×51.

By comparing all GPCR classes, we find that 60 out of 94 determinant positions are unique to one class, whereas 27 are found in two classes and 7 in three classes (Fig. 4b). The determinants common for at least two classes are even fewer when considering their type: ten inactivators (maximum five in A–F), five activators (maximum two in A–B1) and four switches (all between A and B1) (Fig. 4b and Extended Data Fig. 3). These findings demonstrate that although they belong to the same superfamily, use the same structural scaffold and share several macroswitches (helices), the GPCR classes differ in their microswitches (residues). This suggests that there is an ensemble of structural/mechanistic solutions that are available for the GPCR superfamily during evolution and that different receptor classes have explored. It also means that while thinking about developing drugs with different modalities—especially for classes C and F—one should aim to interact with or modulate class-specific state determinants and residue-level microswitches rather than use the same microswitches as in class A.

### State determinants and functional interface sites

**Fig. 6:**
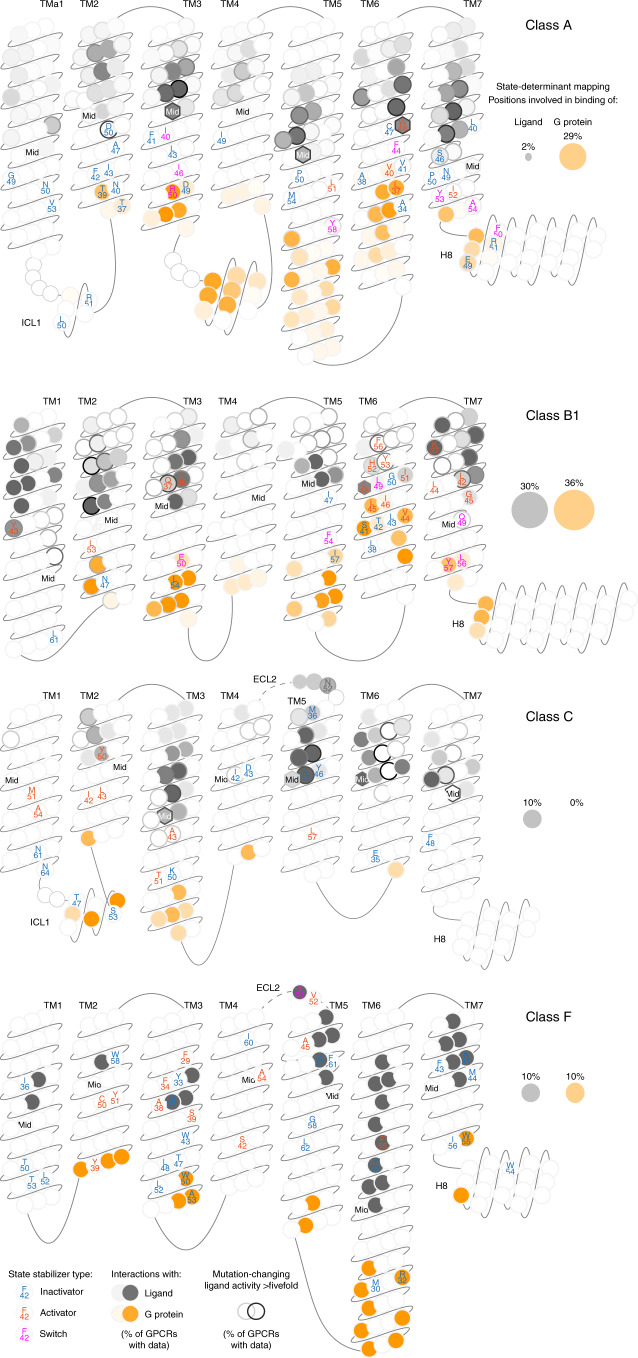
Residue positions stabilizing an inactive and/or active receptor state. GPCR snakeplots mapping the residue positions that form distinct contacts between state determinants classified as inactivators (blue), activators (red) and switches (magenta). Residues are denoted with the consensus amino acid of the investigated receptor structures (Supplementary Table 1) and their generic residue number^26^. Filled positions map ligand- (gray) and G-protein (orange) interaction frequency among all GPCR structures in the given class that have such data (Supplementary Data 2). Allosteric ligand-interacting positions outside of the upper part of the transmembrane helix domain and ECL2 (the orthosteric binding pocket in classes A, B1 and F) are omitted. Border grayscale denotes the frequency of mutations changing the ligand affinity or activity over fivefold. The label ‘Mid’ within hexagon-shaped positions denotes the membrane mid, above and below which the ligand positions are subdivided into ‘upper 7Tm and ECL2’ or ‘other’).

To obtain topological and functional mapping of the state determinants, we mapped their location in relation to ligand and Gα protein-interacting positions from structures (Fig. 6 and Supplementary Data 2). We find that 2%, 10%, 10% and 30% of determinants in classes class A, C, F and B1, respectively, map to ligand-interacting positions in the upper part of the transmembrane helix domain or ECL2 (the orthosteric binding pocket in classes A, B1 and F). The single such determinant in class A is the helix rotation switch W6×48. Furthermore, 0%, 10%, 29% and 36% of determinants in classes class C, F, A and B1, respectively, map to G-protein-interacting positions. Together, this shows that while most determinants are in the transduction pathway between ligand and G-protein sites, except for in class B1, ligands and G proteins can sense and stabilize receptor states by directly interacting with state determinants.

Given their important role in modulating GPCR activity, we specifically mapped switches, that is, determinant residues, that alternate contacts across the inactive/active states. In class A, two switches (R3×50 and 5×58) have direct G-protein interactions, whereas five switches are in transmembrane helices and between ligand and G-protein positions—thereby facilitating signal transduction across the membrane. Class B1 switches are distributed across orthosteric ligand (6×49), G protein (3×50, 7×56 and 7×57) and two other (5×54 and 7×49) positions. Class C uniquely has no switches and the single switch of class F is located in position 45×51, which is a ligand-interacting position in ECL2. These findings show that switches are spread throughout the 7TM bundle. Altogether, the functional mapping may help to explain observed effects on ligand or G-protein affinity from remote mutations, and presents a residue-level rationale for conformational selection^35^ and allosteric modulation^36^.

## Discussion

We present molecular mechanistic maps for activation across the GPCR superfamily and helix macro/microscale residues while extending beyond the transmembrane region to H8 and structurally conserved loop segments. This study has shown that activation of other classes cannot be modeled on the basis of class A. Our findings demonstrate that, although they belong to the same superfamily, use the same structural scaffold and share several helix macroswitches, the GPCR classes differ in their microswitch residue positions, contacts and amino acids. This applies also to class B1, which shares about half of the determinant positions in class A, as the similarity is very small if considering the type of determinant—stabilizing an inactive, active or both states—and their specific consensus amino acids. This highlights the need to elucidate restraints and diverse activation mechanisms separately for each GPCR class and in more detail, to adequately capture structure-function relationships. Determinants of receptor activity are closely tied to the molecular mechanisms of conformational selection^35^ and allosteric modulation^36^, and influence an array of functions and responses, including ligand affinity, basal activity, efficacy and G-protein coupling. Therefore, the contact maps (Fig. 4) presented here provide an actionable foundation for the field, to design and interpret experiments across structural, biophysical (for example, fluorescence and double electron–electron resonance (DEER)), molecular dynamics and mutagenesis studies. The maps also inform drug discovery of which inactivating and activating state determinants are in the orthosteric and allosteric ligand sites and may therefore be exploited to design inverse agonists, neutral antagonists or agonists for different receptors.

The extensive engineering and limited resolution of some structures is an inherent limitation for all structure-based studies. Therefore, more native-like structures and advances in their determination are of utmost value and will serve to continuously refine structure-function relationships generally. For example, the universal TM5 switch reported herein was not discernible until cryo-EM allowed for more structures with a native TM5–ICL3–TM6 region (often subjected to deletion and protein fusion specifically in crystallography). It has furthermore been suggested that there are sequential conformational changes during GPCR activation and G-protein coupling, with transient intermediate states facilitating the transition of the extensive conformational rearrangement, which is not captured by currently available complex structures^37,38^. The proposed intermediate-state complexes may require additional state determinants beyond the ones identified here. Hence, going forward, it will be important to combine structural studies with biophysical investigations, such as fluorescence resonance energy transfer (FRET)-based systems^39^, DEER^40^, NMR^41^ or even mass spectroscopy^42^, for monitoring specific interactions in more infrequent conformations.

Our combined contact frequency and residue-pair conservation cut-offs uniquely address the class representativeness and allow the GPCR superfamily to be described. Classes A, B1, C and F have a 60, 42, 81 and 82% average conservation of determinant consensus amino acids, respectively. For these reasons, the common conserved determinants identified herein should still apply as our knowledge expands from new structures, which could also allow additional determinants to fulfill these cut-offs. Furthermore, although specific receptors and subsets thereof could have additional activation mechanisms not conserved in the whole class, such specific and general mechanisms, respectively, could act in concert.

## Methods

### Structure annotation and selection of representative template dataset

We annotated 510 GPCR structures from the Protein Data Bank^49^. These were all class A and B1 GPCR structures released before 1 November 2020, and all class C and F GPCR structures released by July 2021 (due to the relatively few templates, the two latter classes were updated during manuscript revision). For the two GABAB_1–2_ heterodimer structures (PDB 7C7Q and 7C7S), two additional artificial structures (PDB YZ01 and YZ02, respectively) were used herein to separate the GABA_B1_ monomer from the GABA_B12_ monomer. We selected a representative structure of each GPCR and inactive or active state by applying comprehensive filter criteria spanning completeness of receptor and G protein (≥83% and ≥43% of generic residue positions, respectively), sequence identity >90% to human, resolution ≤3.6 Å, degree active^50^ (≤20% and ≥90% for inactive and active structures, respectively) and consistent ligand modality and state^50^ (inverse agonist/antagonist inactive and agonist active). For representative templates of the active state, a G-protein complex was required. For GABA_B2_–G_i1_ the PDB entry (7C7Q) does not include the G protein and the complex structure model was instead received from the authors (the EM data are deposited in the EMDB database under EMD-30300) (Supplementary Data 1)^46^.

### Transmembrane helix rearrangement analysis

Two-dimensional (2D) plots for TM1–7 segment movement at the extracellular end, cytosolic end and membrane mid, respectively, were produced using our webserver for comparative structure analysis (http://review.gpcrdb.org/structure_comparison/comparative_analysis and ref. ^50^). Class counts of moving TM1–7 were calculated as the sum of consensus movements above 1.0 Å.

### Segment (TM1–7, H8 and loops) contact analysis

Activation-dependent changes in segment networks were determined using 2D network plots of segment contacts^50^. Segment switches (or helix switches) were defined as the segments with distinct contacts (see ‘Generic residue numbering’) in both states.

### Generic residue numbering

Corresponding residue positions in each class were indexed with the structure-based GPCRdb generic residue numbering system^26^. This builds on the sequence-based generic residue numbering systems for classes A (Ballesteros–Weinstein), B1 (Wootten), C (Pin) and F (Wang), but preserves gaps from a structural alignment of two receptors caused by a unique helix bulge or constriction in the sequence alignment, thereby avoiding offset of these and the following residues. All schemes assign residue numbers relative to the most conserved amino acid residue, which is given the number 50, and prefixed with the TM helix number (for example, 3×32 is on TM3 and 18 positions before the reference). This generic residue numbering scheme also uniquely indexes H8 and structurally conserved loop segments, which are numbered by the preceding and following TM helix (for example, 45 is ECL2 located between TM4 and TM5).

### State-stabilizing contact identification

State-stabilizing contacts were identified using the Structure comparison tool^50^. The most distinct contact in class A (1×49–7×50 contact) has 80% higher frequency in the inactive than in the active state, and the specificity of state-specific contacts depends on the number of members and templates in each class. To obtain comparable numbers of networks and contacts, we therefore adjusted the class frequency difference thresholds accordingly (no. members and inactive/active-state templates): A: 40% (285, 33/14), B1: 67% (15, 3/10), C: 75% (22, 4/2) and F: 100% (11, 2/2). To ensure that the identified determinants have a wide role in each class, we also applied a sequence conservation cut-off. This cut-off requires the amino acids pairs that make up a state-specific contact to be conserved, and therefore able to be formed in at least 30% of all receptors in the given GPCR class. The remaining residues, referred to as ‘state determinants’ or ‘state stabilizers’, were further classified into ‘inactivators’, ‘activators’ and ‘switches’ on the basis of whether their most frequent contact occurs in inactive, active and both states, respectively.

### State-determinant topological mapping in relation to ligand and G-protein sites

We mapped state stabilizers to functional sites to ligand and G-protein sites using the comparative structure analysis tool of GPCRdb^50^. Allosteric ligand-interacting positions outside the upper part of the transmembrane helix domain and ECL2 (the orthosteric binding pocket in classes A, B1 and F) were omitted.

### Mutagenesis and BRET-based signaling assay

Codon-optimized human β_2_-adrenoceptor (β_2_) was cloned into pcDNA3.1 with an N-terminal signal sequence, Twin-Strep-tag and SNAP-tag. All biosensor constructs were in pcDNA3.1 (G_15_ biosensor^51^ and G_s_ sensor:^52,53^). Mutations were made as described in ref. ^54^. Biosensor and receptor DNA was transiently transfected into HEKSL cells (a gift from S. Laporte). Cell culture and transfection was performed as described in ref. ^55^. After incubation for two days at 37 °C with 5% CO_2_, DMEM was replaced with Tyrode’s buffer (137 mM NaCl, 0.9 mM KCl, 1 mM MgCl_2_, 11.9 mM NaHCO_3_, 3.6 mM NaH_2_PO_4_, 25 mM HEPES, 5.5 mM glucose,1 mM CaCl_2_, pH 7.4), followed by incubation for at least 30 min at 37 °C. Ligand was added 10 min before the measurement and the luciferase substrate coelenterazine 400a (Nanolight Technology) was added 5 min before the measurement. Coelenterazine 400a was added to a final concentration of 5 μM and ligand concentrations ranged from 31.6 nM to 3.16 mM in half-log steps. In addition, a buffer control was included. BRET was read in a Synergy Neo (Biotek) plate reader at 410 and 515 nm. All signaling experiments were done in biological triplicates.

### Statistics

For the experimental alanine mutations of predicted state-changing and nonstate-changing residues (Fig. 5), statistical significance has been assessed by a two-sided Wilcoxon rank-sum test (*n* = 6 for each category, individual data points in Supplementary Table 2).

### Reporting Summary

Further information on research design is available in the Nature Research Reporting Summary linked to this article.

## Online content

Any methods, additional references, Nature Research reporting summaries, source data, extended data, supplementary information, acknowledgements, peer review information; details of author contributions and competing interests; and statements of data and code availability are available at 10.1038/s41594-021-00674-7.

## Supplementary information

Supplementary Information Supplementary Tables 1 and 2.

Reporting Summary

Peer Review Information

Supplementary Data 1 GPCR structure annotation to define activation states and representative templates. Excel file containing annotation of all 510 GPCR structures from the Protein Data Bank^65^. These are all class A and B1 GPCR structures released before 1 November 2020, and all class C and F GPCR structures released by July 2021 (due to the relatively few templates, the two latter classes were updated during manuscript revision). For the two GABAB_1–2_ heterodimer structures (PDB: 7C7Q and 7C7S), two additional artificial structures (PDB: YZ01 and YZ02, respectively) were used herein to separate the GABA_B1_ monomer from the GABA_B12_ monomer. Representative structures of each GPCR and inactive or active state were selected by applying comprehensive filter criteria spanning completeness of receptor and G protein (≥86% and ≥43% of generic residue positions, respectively), sequence identity >90% to human, resolution ≤4.0 Å, degree active (≤20% and ≥90% for inactive and active structures, respectively) and consistent ligand modality and state (inverse agonist/antagonist inactive and agonist active). A continuous annotation is provided in the GPCRdb structure browser (http://review.gpcrdb.org/structure) and the structure comparison webserver (Extended Data Fig. 1).

Supplementary Data 2 Topological and functional mapping of the state-determinant positions. Mapping of state-determinant 309 ligand and 103 G-protein-interacting positions (gray and orange fill, respectively) extracted here from 423 GPCR crystal and cryo-EM structures. Each residue position has been assigned one main role defined by ligand and G-protein-interacting positions located within the intracellular and extracellular TM helix bundle pockets, respectively, and signal transduction pathway residue bridging such positions on the same helix in at least two classes (Methods).

## Data Availability

All data are available in GPCRdb (https://review.gpcrdb.org), GitHub (https://github.com/protwis/gpcrdb_data) and Supplementary Data 1 and 2.

## Code availability

No code was developed for this manuscript, which instead used the existing GPCRdb^56^ resources, including a new comparative structure analysis platform^50^. All open-source code can be obtained from GitHub (https://github.com/protwis/protwis) under the permissive Apache 2.0 License (https://www.apache.org/licenses/LICENSE-2.0). A complete list of the software used for data analysis is available from the Nature Research Reporting Summary.
