# Supplementary material for: GPCR activation mechanisms across classes and macro/microscales
Source: Nat Struct Mol Biol. 2021 Nov 10;28(11):879–88. doi: 10.1038/s41594-021-00674-7 (PMC8580822; doi:10.1038/s41594-021-00674-7)
Supplement: Supplementary file 1 — Supplementary Tables 1 and 2. [file 41594_2021_674_MOESM1_ESM.pdf]

nature portfolio

https://doi.org/10.1038/s41594-021-00674-7

Supplementary information

GPCR activation mechanisms across classes and macro/microscales

In the format provided by the authors and unedited

Supplementary Table 1 | Representative structures of GPCRs and inactive and active states used as templates.

The structure annotation and criteria-based filtering to select these templates is shown in Supplementary Spreadsheet 1.

Cl	Receptor family	UniProt	IUPHAR	Inactive PDB ID	Active PDB ID	Inactive Ref.	Active Ref.
A	Adrenoceptors	ADA2B	α_{2B}		6K41		1
A	Cannabinoid	CNR1	CB_1		6N4B		2
A	Chemokine	CCR6	CCR6		6WWZ		3
A	Formylpeptide	FPR2	FPR2/ALX		6OMM		4
A	Neurotensin	NTR1	NTS1		6OS9		5
A	Adrenoceptors	ADRB2	β_2	2RH1	3SN6	6	7
A	Dopamine	DRD3	D_3	3PBL		8	
A	Histamine	HRH1	H_1	3RZE		9	
A	Lysophospholipid (S1P)	S1PR1	$S1P_1$	3V2Y		10	
A	Opioid	OPRK	κ	4DJH		11	
A	Opioid	OPRM	μ	4DKL	6DDE	12	13
A	Opioid	OPRD	δ	4N6H		14	
A	Acetylcholine (muscarinic)	ACM3	M_3	4U15		15	
A	Lysophospholipid (LPA)	LPAR1	LPA_1	4Z36		16	
A	Angiotensin	AGTR1	AT_1	4ZUD		17	
A	Acetylcholine (muscarinic)	ACM1	M_1	5CXV	6OIJ	18	19
A	Opioid	OPRX	NOP	5DHH		20	
A	Acetylcholine (muscarinic)	ACM4	M_4	5DSG		18	
A	Adenosine	AA2AR	A_{2A}	5NM4	5G53	21	22
A	Adenosine	AA1R	A_1	5UEN	6D9H	23	24
A	Dopamine	DRD4	D_4	5WIU		25	
A	Orexin	OX2R	OX_2	5WQC		26	
A	Endothelin	EDNRB	ETB	5X93		27	
A	Neuropeptide Y	NPY1R	Y_1	5ZBQ		28	
A	Acetylcholine (muscarinic)	ACM2	M_2	5ZKC	6OIK	29	19
A	Cannabinoid	CNR2	CB_2	5ZTY	6KPF	30	31
A	5-Hydroxytryptamine	5HT2A	$5\text{-}HT_{2A}$	6A94		32	
A	5-Hydroxytryptamine	5HT2C	$5\text{-}HT_{2C}$	6BQH		33	
A	Complement peptide	C5AR1	$C5a_1$	6C1R		34	
A	Dopamine	DRD2	D_2	6CM4		35	
A	Tachykinin	NK1R	NK_1	6HLP		36	
A	Ghrelin	GHSR	Ghrelin	6KO5		37	
A	Adrenoceptors	ADA2C	α_{2C}	6KUW		38	
A	Chemokine	CXCR2	CXCR2	6LFL	6LFO	39	39
A	Acetylcholine (muscarinic)	ACM5	M_5	6OL9		40	
A	Melatonin	MTR1A	MT_1	6PS8		41	
A	Orexin	OX1R	OX_1	6TOD		42	
A	Vasopressin and oxytocin	OXYR	OT	6TPK		43	
B1	Calcitonin	CALCR	CT		6NIY		44
B1	Calcitonin	CALRL	Calcitonin-like		6UVA		45
B1	Corticotropin-releasing factor	CRFR2	CRF2		6PB1		46
B1	Glucagon	SCTR	Secretin		6WZG		47
B1	VIP and PACAP	PACR	PAC1		6M1I		48
B1	VIP and PACAP	VIPR1	VPAC1		6VN7		49
B1	Corticotropin-releasing factor	CRFR1	CRF1	4K5Y	6P9X	46	50
B1	Glucagon	GLR	Glucagon	5EE7	6WPW	51	52
B1	Parathyroid hormone	PTH1R	PTH1		6NBH		53
B1	Glucagon	GLP1R	GLP-1	6LN2	6X18	54	55
C	Metabotropic glutamate	GRM1	$mGlu_1$	4OR2		56	
C	Metabotropic glutamate	GRM2	$mGlu_2$		7E9G		57
C	Metabotropic glutamate	GRM5	$mGlu_5$	6FFI		58	
C	GABAB	GABR1	$GABA_{B1}$	7C7S		59	
C	GABAB	GABR2	$GABA_{B2}$	7C7S	7C7Q	59	59
F	Frizzled	SMO	SMO	4JKV	6XBK	60	61
F	Frizzled	FZD4	FZD_4	6BD4		62	
F	Frizzled	FZD7	FZD_7	7EVW			63

Supplementary Table 2 | State-changing residue mutation potency and Emax values. Individual data points for the potency reduction and Emax values plotted and visualised in Figure 5.

Generic residue number	State contact difference	Gs pEC50 reduction (log units)	G15 pEC50 reduction (log units)	Gs Emax (%)	G15 Emax (%)
7x50	151	1.2	1.5	83	75
5x58	92	2.0	1.7	93	17
23x52	90	0.5	1.1	50	58
7x46	86	0.8	1.1	107	90
2x42	84	1.1	1.2	60	77
3x41	81	0.8	0.9	117	101
1x51	1	-0.2	0.1	99	99
3x42	1	0.0	0.2	47	39
4x51	1	0.4	0.4	85	91
4x52	1	0.7	0.6	76	93
6x61	1	-0.4	-0.1	102	98
3x51	0	0.8	0.3	73	87

References

1 Yuan, D. *et al.* Activation of the alpha2B adrenoceptor by the sedative sympatholytic dexmedetomidine. *Nat. Chem. Biol.* **16**, 507-512, doi:10.1038/s41589-020-0492-2 (2020).

2 Krishna Kumar, K. *et al.* Structure of a Signaling Cannabinoid Receptor 1-G Protein Complex. *Cell* **176**, 448-458 e412, doi:10.1016/j.cell.2018.11.040 (2019).

3 Wasilko, D. J. *et al.* Structural basis for chemokine receptor CCR6 activation by the endogenous protein ligand CCL20. *Nat Commun* **11**, 3031, doi:10.1038/s41467-020-16820-6 (2020).

4 Zhuang, Y. *et al.* Structure of formylpeptide receptor 2-Gi complex reveals insights into ligand recognition and signaling. *Nat Commun* **11**, 885, doi:10.1038/s41467-020-14728-9 (2020).

5 Kato, H. E. *et al.* Conformational transitions of a neurotensin receptor 1-Gi1 complex. *Nature* **572**, 80-85, doi:10.1038/s41586-019-1337-6 (2019).

6 Cherezov, V. *et al.* High-resolution crystal structure of an engineered human beta2-adrenergic G protein-coupled receptor. *Science* **318**, 1258-1265, doi:10.1126/science.1150577 (2007).

7 Rasmussen, S. G. *et al.* Crystal structure of the beta2 adrenergic receptor-Gs protein complex. *Nature* **477**, 549-555, doi:10.1038/nature10361 (2011).

8 Chien, E. Y. *et al.* Structure of the human dopamine D3 receptor in complex with a D2/D3 selective antagonist. *Science* **330**, 1091-1095, doi:10.1126/science.1197410 (2010).

9 Shimamura, T. *et al.* Structure of the human histamine H1 receptor complex with doxepin. *Nature* **475**, 65-70, doi:10.1038/nature10236 (2011).

10 Hanson, M. A. *et al.* Crystal structure of a lipid G protein-coupled receptor. *Science* **335**, 851-855, doi:10.1126/science.1215904 (2012).

11 Wu, H. *et al.* Structure of the human kappa-opioid receptor in complex with JDTic. *Nature* **485**, 327-332, doi:10.1038/nature10939 (2012).

12 Manglik, A. *et al.* Crystal structure of the micro-opioid receptor bound to a morphinan antagonist. *Nature* **485**, 321-326, doi:10.1038/nature10954 (2012).

13 Koehl, A. *et al.* Structure of the micro-opioid receptor-Gi protein complex. *Nature* **558**, 547-552, doi:10.1038/s41586-018-0219-7 (2018).

14 Fenalti, G. *et al.* Molecular control of delta-opioid receptor signalling. *Nature* **506**, 191-196, doi:10.1038/nature12944 (2014).

15 Thorsen, T. S., Matt, R., Weis, W. I. & Kobilka, B. K. Modified T4 Lysozyme Fusion Proteins Facilitate G Protein-Coupled Receptor Crystallogenesis. *Structure* **22**, 1657-1664, doi:10.1016/j.str.2014.08.022 (2014).

16 Chrencik, J. E. *et al.* Crystal Structure of Antagonist Bound Human Lysophosphatidic Acid Receptor 1. *Cell* **161**, 1633-1643, doi:10.1016/j.cell.2015.06.002 (2015).

17 Zhang, H. *et al.* Structural Basis for Ligand Recognition and Functional Selectivity at Angiotensin Receptor. *The Journal of biological chemistry* **290**, 29127-29139, doi:10.1074/jbc.M115.689000 (2015).

18 Thal, D. M. *et al.* Crystal structures of the M1 and M4 muscarinic acetylcholine receptors. *Nature* **531**, 335-340, doi:10.1038/nature17188 (2016).

19 Maeda, S., Qu, Q., Robertson, M. J., Skiniotis, G. & Kobilka, B. K. Structures of the M1 and M2 muscarinic acetylcholine receptor/G-protein complexes. *Science* **364**, 552-557, doi:10.1126/science.aaw5188 (2019).

20 Miller, R. L. *et al.* The Importance of Ligand-Receptor Conformational Pairs in Stabilization: Spotlight on the N/OFQ G Protein-Coupled Receptor. *Structure* **23**, 2291-2299, doi:10.1016/j.str.2015.07.024 (2015).

21 Weinert, T. *et al.* Serial millisecond crystallography for routine room-temperature structure determination at synchrotrons. *Nat Commun* **8**, 542, doi:10.1038/s41467-017-00630-4 (2017).

22 Carpenter, B., Nehme, R., Warne, T., Leslie, A. G. & Tate, C. G. Structure of the adenosine A(2A) receptor bound to an engineered G protein. *Nature* **536**, 104-107, doi:10.1038/nature18966 (2016).

23 Glukhova, A. *et al.* Structure of the Adenosine A1 Receptor Reveals the Basis for Subtype Selectivity. *Cell* **168**, 867-877 e813, doi:10.1016/j.cell.2017.01.042 (2017).

24 Draper-Joyce, C. J. *et al.* Structure of the adenosine-bound human adenosine A1 receptor-Gi complex. *Nature* **558**, 559-563, doi:10.1038/s41586-018-0236-6 (2018).

25 Wang, S. *et al.* D4 dopamine receptor high-resolution structures enable the discovery of selective agonists. *Science* **358**, 381-386, doi:10.1126/science.aan5468 (2017).

26 Suno, R. *et al.* Crystal Structures of Human Orexin 2 Receptor Bound to the Subtype-Selective Antagonist EMPA. *Structure* **26**, 7-19 e15, doi:10.1016/j.str.2017.11.005 (2018).

27 Shihoya, W. *et al.* X-ray structures of endothelin ETB receptor bound to clinical antagonist bosentan and its analog. *Nat. Struct. Mol. Biol.* **24**, 758-764, doi:10.1038/nsmb.3450 (2017).

28 Yang, Z. *et al.* Structural basis of ligand binding modes at the neuropeptide Y Y1 receptor. *Nature* **556**, 520-524, doi:10.1038/s41586-018-0046-x (2018).

29 Suno, R. *et al.* Structural insights into the subtype-selective antagonist binding to the M2 muscarinic receptor. *Nat. Chem. Biol.* **14**, 1150-1158, doi:10.1038/s41589-018-0152-y (2018).

30 Li, X. *et al.* Crystal Structure of the Human Cannabinoid Receptor CB2. *Cell* **176**, 459-467 e413, doi:10.1016/j.cell.2018.12.011 (2019).

31 Hua, T. *et al.* Activation and Signaling Mechanism Revealed by Cannabinoid Receptor-Gi Complex Structures. *Cell* **180**, 655-665 e618, doi:10.1016/j.cell.2020.01.008 (2020).

32 Kimura, K. T. *et al.* Structures of the 5-HT2A receptor in complex with the antipsychotics risperidone and zotepine. *Nat. Struct. Mol. Biol.* **26**, 121-128, doi:10.1038/s41594-018-0180-z (2019).

33 Peng, Y. *et al.* 5-HT2C Receptor Structures Reveal the Structural Basis of GPCR Polypharmacology. *Cell* **172**, 719-730 e714, doi:10.1016/j.cell.2018.01.001 (2018).

34 Liu, H. *et al.* Orthosteric and allosteric action of the C5a receptor antagonists. *Nat. Struct. Mol. Biol.* **25**, 472-481, doi:10.1038/s41594-018-0067-z (2018).

35 Wang, S. *et al.* Structure of the D2 dopamine receptor bound to the atypical antipsychotic drug risperidone. *Nature* **555**, 269-273, doi:10.1038/nature25758 (2018).

36 Schoppe, J. *et al.* Crystal structures of the human neurokinin 1 receptor in complex with clinically used antagonists. *Nat Commun* **10**, 17, doi:10.1038/s41467-018-07939-8 (2019).

37 Shiimura, Y. *et al.* Structure of an antagonist-bound ghrelin receptor reveals possible ghrelin recognition mode. *Nat Commun* **11**, 4160, doi:10.1038/s41467-020-17554-1 (2020).

38 Chen, X. *et al.* Molecular Mechanism for Ligand Recognition and Subtype Selectivity of alpha2C Adrenergic Receptor. *Cell reports* **29**, 2936-2943 e2934, doi:10.1016/j.celrep.2019.10.112 (2019).

39 Liu, K. *et al.* Structural basis of CXC chemokine receptor 2 activation and signalling. *Nature* **585**, 135-140, doi:10.1038/s41586-020-2492-5 (2020).

40 Vuckovic, Z. *et al.* Crystal structure of the M5 muscarinic acetylcholine receptor. *Proceedings of the National Academy of Sciences of the United States of America* **116**, 26001-26007, doi:10.1073/pnas.1914446116 (2019).

41 Ishchenko, A. *et al.* Toward G protein-coupled receptor structure-based drug design using X-ray lasers. *IUCrJ* **6**, 1106-1119, doi:10.1107/S2052252519013137 (2019).

42 Rappas, M. *et al.* Comparison of Orexin 1 and Orexin 2 Ligand Binding Modes Using X-ray Crystallography and Computational Analysis. *Journal of medicinal chemistry* **63**, 1528-1543, doi:10.1021/acs.jmedchem.9b01787 (2020).

43 Waltenspuhl, Y., Schoppe, J., Ehrenmann, J., Kummer, L. & Pluckthun, A. Crystal structure of the human oxytocin receptor. *Sci Adv* **6**, eabb5419, doi:10.1126/sciadv.abb5419 (2020).

44 Dal Maso, E. *et al.* The Molecular Control of Calcitonin Receptor Signaling. *ACS Pharmacol Transl Sci* **2**, 31-51, doi:10.1021/acsptsci.8b00056 (2019).

45 Liang, Y. L. *et al.* Structure and Dynamics of Adrenomedullin Receptors AM1 and AM2 Reveal Key Mechanisms in the Control of Receptor Phenotype by Receptor Activity-Modifying Proteins. *ACS Pharmacol Transl Sci* **3**, 263-284, doi:10.1021/acsptsci.9b00080 (2020).

46 Hollenstein, K. *et al.* Structure of class B GPCR corticotropin-releasing factor receptor 1. *Nature* **499**, 438-443, doi:10.1038/nature12357 (2013).

47 Dong, M. *et al.* Structure and dynamics of the active Gs-coupled human secretin receptor. *Nat Commun* **11**, 4137, doi:10.1038/s41467-020-17791-4 (2020).

48 Wang, J. *et al.* Cryo-EM structures of PAC1 receptor reveal ligand binding mechanism. *Cell Res.* **30**, 436-445, doi:10.1038/s41422-020-0280-2 (2020).

49 Duan, J. *et al.* Cryo-EM structure of an activated VIP1 receptor-G protein complex revealed by a NanoBiT tethering strategy. *Nat Commun* **11**, 4121, doi:10.1038/s41467-020-17933-8 (2020).

50 Liang, Y. L. *et al.* Toward a Structural Understanding of Class B GPCR Peptide Binding and Activation. *Mol. Cell* **77**, 656-668 e655, doi:10.1016/j.molcel.2020.01.012 (2020).

51 Jazayeri, A. *et al.* Extra-helical binding site of a glucagon receptor antagonist. *Nature* **533**, 274-277, doi:10.1038/nature17414 (2016).

52 Hilger, D. *et al.* Structural insights into differences in G protein activation by family A and family B GPCRs. *Science* **369**, eaba3373, doi:10.1126/science.aba3373 (2020).

53 Zhao, L. H. *et al.* Structure and dynamics of the active human parathyroid hormone receptor-1. *Science* **364**, 148-153, doi:10.1126/science.aav7942 (2019).

54 Wu, F. *et al.* Full-length human GLP-1 receptor structure without orthosteric ligands. *Nat Commun* **11**, 1272, doi:10.1038/s41467-020-14934-5 (2020).

55 Zhang, X. *et al.* Differential GLP-1R Binding and Activation by Peptide and Non-peptide Agonists. *Mol. Cell* **80**, 485-500 e487, doi:10.1016/j.molcel.2020.09.020 (2020).

56 Wu, H. *et al.* Structure of a class C GPCR metabotropic glutamate receptor 1 bound to an allosteric modulator. *Science* **344**, 58-64, doi:10.1126/science.1249489 (2014).

57 Lin, S. *et al.* Structures of G(i)-bound metabotropic glutamate receptors mGlu2 and mGlu4. *Nature* **594**, 583-588, doi:10.1038/s41586-021-03495-2 (2021).

58 Christopher, J. A. *et al.* Structure-Based Optimization Strategies for G Protein-Coupled Receptor (GPCR) Allosteric Modulators: A Case Study from Analyses of New Metabotropic Glutamate Receptor 5 (mGlu5) X-ray Structures. *Journal of medicinal chemistry* **62**, 207-222, doi:10.1021/acs.jmedchem.7b01722 (2019).

59 Mao, C. *et al.* Cryo-EM structures of inactive and active GABAB receptor. *Cell Res.* **30**, 564-573, doi:10.1038/s41422-020-0350-5 (2020).

60 Wang, C. *et al.* Structure of the human smoothened receptor bound to an antitumour agent. *Nature* **497**, 338-343, doi:10.1038/nature12167 (2013).

61 Qi, X., Friedberg, L., De Bose-Boyd, R., Long, T. & Li, X. Sterols in an intramolecular channel of Smoothened mediate Hedgehog signaling. *Nat. Chem. Biol.* **16**, 1368-1375, doi:10.1038/s41589-020-0646-2 (2020).

62 Yang, S. *et al.* Crystal structure of the Frizzled 4 receptor in a ligand-free state. *Nature* **560**, 666-670, doi:10.1038/s41586-018-0447-x (2018).

63 . doi:10.1038/s41422-021-00525-6 (
