# Supplementary material for: GPCR activation mechanisms across classes and macro/microscales
Source: Nat Struct Mol Biol. 2021 Nov 10;28(11):879–88. doi: 10.1038/s41594-021-00674-7 (PMC8580822; doi:10.1038/s41594-021-00674-7)
Supplement: Supplementary file 2 — Reporting Summary [file 41594_2021_674_MOESM2_ESM.pdf]

nature research

Corresponding author(s): David E. Gloriam

Last updated by author(s): Sep 9, 2021

Reporting Summary

Nature Research wishes to improve the reproducibility of the work that we publish. This form provides structure for consistency and transparency in reporting. For further information on Nature Research policies, see our Editorial Policies and the Editorial Policy Checklist.

Statistics

For all statistical analyses, confirm that the following items are present in the figure legend, table legend, main text, or Methods section.

n/a	Confirmed	
☐	☒	The exact sample size (*n*) for each experimental group/condition, given as a discrete number and unit of measurement
☒	☐	A statement on whether measurements were taken from distinct samples or whether the same sample was measured repeatedly
☐	☒	The statistical test(s) used AND whether they are one- or two-sided<br>*Only common tests should be described solely by name; describe more complex techniques in the Methods section.*
☒	☐	A description of all covariates tested
☒	☐	A description of any assumptions or corrections, such as tests of normality and adjustment for multiple comparisons
☐	☒	A full description of the statistical parameters including central tendency (e.g. means) or other basic estimates (e.g. regression coefficient) AND variation (e.g. standard deviation) or associated estimates of uncertainty (e.g. confidence intervals)
☐	☒	For null hypothesis testing, the test statistic (e.g. *F*, *t*, *r*) with confidence intervals, effect sizes, degrees of freedom and *P* value noted<br>*Give P values as exact values whenever suitable.*
☒	☐	For Bayesian analysis, information on the choice of priors and Markov chain Monte Carlo settings
☐	☒	For hierarchical and complex designs, identification of the appropriate level for tests and full reporting of outcomes
☒	☐	Estimates of effect sizes (e.g. Cohen's *d*, Pearson's *r*), indicating how they were calculated

Our web collection on statistics for biologists contains articles on many of the points above.

Software and code

Policy information about availability of computer code

Data collection	GPCRdb source code: https://github.com/protwis/protwis
Data analysis	GPCRdb web resource: https://gpcrdb.org/ (release November 2020)<br>Microsoft Office 365: www.microsoft.com (version 16.52)<br>GraphPad Prism: https://www.graphpad.com/scientific-software/prism/

For manuscripts utilizing custom algorithms or software that are central to the research but not yet described in published literature, software must be made available to editors and reviewers. We strongly encourage code deposition in a community repository (e.g. GitHub). See the Nature Research guidelines for submitting code & software for further information.

Data

Policy information about availability of data

All manuscripts must include a data availability statement. This statement should provide the following information, where applicable:

- Accession codes, unique identifiers, or web links for publicly available datasets
- A list of figures that have associated raw data
- A description of any restrictions on data availability

All relevant data are integrated into the web resource in the GPCR database at http://www.gpcrdb.org) and are available at GitHub (https://github.com/protwis/gpcrdb_data). All other data that support the findings of this study are provided as Extended Data or Supplementary Information. This study used data from the Guide to Pharmacology database (https://www.guidetopharmacology.org/webServices.jsp, release June 2017) and RCSB Protein Data Bank (https://www.rcsb.org, last release in July 2021). PDB codes: 2RH1, 3PBL, 3RZE, 3SN6, 3V2Y, 4DJH, 4DKL, 4JKV, 4K5Y, 4N6H, 4OR2, 4U15, 4Z36, 4ZUD, 5CXV, 5DHH, 5DSG, 5EE7, 5G53, 5NM4, 5UEN, 5WIU, 5WQC, 5X93, 5ZBQ, 5ZKC, 5ZTY, 6A94, 6BD4, 6BQH, 6C1R, 6CM4, 6D9H, 6DDE, 6FFI, 6HLP, 6K41, 6KO5, 6KPF, 6KUW, 6LFL, 6LFO, 6LN2, 6M1I,

6N4B, 6NBH, 6NIY, 6OIJ, 6OIK, 6OL9, 6OMM, 6OS9, 6P9X, 6PB1, 6PS8, 6TOD, 6TPK, 6UVA, 6VN7, 6WPW, 6WWZ, 6WZG, 6X18, 6XBK, 7C7Q, 7C7S, 7C7S, 7E9G, and 7EVW.

Field-specific reporting

Please select the one below that is the best fit for your research. If you are not sure, read the appropriate sections before making your selection.

☒ Life sciences ☐ Behavioural & social sciences ☐ Ecological, evolutionary & environmental sciences

For a reference copy of the document with all sections, see nature.com/documents/nr-reporting-summary-flat.pdf

Life sciences study design

All studies must disclose on these points even when the disclosure is negative.

Sample size	We maximised the sample (crystal and cryo-EM structures) coverage of as many receptors and inactive and active states as possible. To compare inactive and active states in each GPCR class one needs at least one receptor in each state. We have more than that and describe the number of structural templates and their distribution across the GPCR classes and states in the manuscript and Extended Data Table 1.'<br><br>For Figure 5, evaluating the effect of mutations on activation state, the statistical significance has been assessed by a two-sided Wilcoxon rank-sum test (n=6 for each category, individual data points in Extended Data Table 2).
Data exclusions	As defined in Methods and Supplementary Spreadsheet 1, we used predefined criteria to exclude GPCR structures not suitable as templates.
Replication	During the course of the study, new structural templates emerged and were added. In two rounds of updates, all values were updated and results obtained were similar (e.g. only few state-specific contacts changed) and none of the overall conclusions/findings in the manuscript changed. All signalling experiments, evaluating mutations of state determinants, were done in biological triplicates.
Randomization	Not applicable. The representative templates for the structural analyses were selected based on quality criteria (see Methods and Supplemental Spreadsheet 1). The mutilations were selected base on the highest and lowest predicted effect on a GPCR activation state.
Blinding	Blinding was not possible because of the selection and analysis of samples (structures) that required intervention.

Reporting for specific materials, systems and methods

We require information from authors about some types of materials, experimental systems and methods used in many studies. Here, indicate whether each material, system or method listed is relevant to your study. If you are not sure if a list item applies to your research, read the appropriate section before selecting a response.

Materials & experimental systems

n/a	Involved in the study	
☒	☐	Antibodies
☐	☒	Eukaryotic cell lines
☒	☐	Palaeontology and archaeology
☒	☐	Animals and other organisms
☒	☐	Human research participants
☒	☐	Clinical data
☒	☐	Dual use research of concern

Methods

n/a	Involved in the study	
☒	☐	ChIP-seq
☒	☐	Flow cytometry
☒	☐	MRI-based neuroimaging

Eukaryotic cell lines

Policy information about cell lines

Cell line source(s)	The HEK293T cells were originally obtained from CTTC and have been maintained in the Bouvier laboratory to develop BRET-based biosensors. The HEK293SL cell line is a subclone of HEK293T cells. This cell line was used for all the BRET experiments performed in the present study.
Authentication	No formal cell line authentication was carried out. The identity of the cells is simply verified by visual examination
Mycoplasma contamination	All cells were regularly tested for mycoplasma contamination (PCR Mycoplasma Detection kit, abm, BC, Canada). Only mycoplasm-negative cell ewer used.
Commonly misidentified lines<br>(See ICLAC register)	No misidentified cell lines were used in this study.
