# Supplementary material for: GPCR activation mechanisms across classes and macro/microscales
Source: Nat Struct Mol Biol. 2021 Nov 10;28(11):879–88. doi: 10.1038/s41594-021-00674-7 (PMC8580822; doi:10.1038/s41594-021-00674-7)
Supplement: Supplementary file 3 — Peer Review Information [file 41594_2021_674_MOESM3_ESM.pdf]

natureresearch

Peer Review Information

Journal: Nature Structural and Molecular Biology
Manuscript Title: GPCR activation mechanisms across classes and macro/microscales
Corresponding author name(s): David Gloriam

Reviewer Comments & Decisions:

Decision Letter, initial version:

2nd Jun 2021

Dear David,

Thank you again for submitting your manuscript "GPCR activation mechanisms across classes and macro/microscales". I apologize for the delay in responding, which, as you already know, resulted from the difficulty in obtaining suitable referee reports. Nevertheless, we now have comments (below) from the 2 reviewers who evaluated your paper. In light of those reports, we remain interested in your study and would like to see your response to the comments of the referees, in the form of a revised manuscript.

You will see that the reviewers (both experts in GPCR structure/function) are positive about the interest and quality of the study, and reviewer 2 recommends publication as is. However, reviewer 1 made several useful suggestions to improve the presentation of the findings and requested experimental validation of key results, which we agree would greatly strengthen the manuscript.

We are committed to providing a fair and constructive peer-review process. Do not hesitate to contact us if there are specific requests from the reviewers that you believe are technically impossible or unlikely to yield a meaningful outcome.

Please be sure to address all concerns of the referees in full in a point-by-point response and highlight all changes in the revised manuscript text file. If you have comments that are intended for editors only, please include those in a separate cover letter.

We expect to see your revised manuscript within 12 weeks. If you cannot send it within this time, please contact us to discuss an extension; we would still consider your revision, provided that no similar work has been accepted for publication at NSMB or published elsewhere.

As you already know, we put great emphasis on ensuring that the methods and statistics reported in our papers are correct and accurate. As such, if there are any changes that should be reported, please submit an updated version of the Reporting Summary along with your revision.

Please follow the links below to download these files:

Reporting Summary:
https://www.nature.com/documents/nr-reporting-summary.pdf

Please note that the form is a dynamic 'smart pdf' and must therefore be downloaded and completed in Adobe Reader.

When submitting the revised version of your manuscript, please pay close attention to our href="https://www.nature.com/nature-research/editorial-policies/image-integrity">Digital Image Integrity Guidelines.

Finally, please ensure that you retain unprocessed data and metadata files after publication, ideally archiving data in perpetuity, as these may be requested during the peer review and production process or after publication if any issues arise.

SOURCE DATA: we urge authors to provide, in tabular form, the data underlying the graphical representations used in figures. This is to further increase transparency in data reporting, as detailed in this editorial (http://www.nature.com/nsmb/journal/v22/n10/full/nsmb.3110.html). Spreadsheets can be submitted in excel format. Only one (1) file per figure is permitted; thus, for multi-paneled figures, the source data for each panel should be clearly labeled in the Excel file; alternately the data can be provided as multiple, clearly labeled sheets in an Excel file. When submitting files, the title field should indicate which figure the source data pertains to. We encourage our authors to provide source data at the revision stage, so that they are part of the peer-review process.

Data availability: this journal strongly supports public availability of data. All data used in accepted papers should be available via a public data repository, or alternatively, as Supplementary Information. If data can only be shared on request, please explain why in your Data Availability Statement, and also in the correspondence with your editor. Please note that for some data types, deposition in a public repository is mandatory - more information on our data deposition policies and available repositories can be found below:
https://www.nature.com/nature-research/editorial-policies/reporting-standards#availability-of-data

While we encourage the use of color in preparing figures, please note that this will incur a charge to partially defray the cost of printing. Information about color charges can be found at
http://www.nature.com/nsmb/authors/submit/index.html#costs

Nature Structural & Molecular Biology is committed to improving transparency in authorship. As part of our efforts in this direction, we are now requesting that all authors identified as 'corresponding author' on published papers create and link their Open Researcher and Contributor Identifier (ORCID) with their account on the Manuscript Tracking System (MTS), prior to acceptance. This applies to primary research papers only. ORCID helps the scientific community achieve unambiguous attribution of all scholarly contributions. You can create and link your ORCID from the home page of the MTS by

clicking on ‘Modify my Springer Nature account’. For more information please visit please visit www.springernature.com/orcid.

Please use the link below to submit your revised manuscript and related files:

[READACTED]

Note: This URL links to your confidential home page and associated information about manuscripts you may have submitted, or that you are reviewing for us. If you wish to forward this email to co-authors, please delete the link to your homepage.

We look forward to seeing the revised manuscript and thank you for the opportunity to review your work.

Kind regards,
Florian

Florian Ullrich, Ph.D.
Associate Editor
Nature Structural & Molecular Biology
ORCID 0000-0002-1153-2040

Reviewers' Comments:

Reviewer #1:
Remarks to the Author:
Hauser et al. used a newly developed online GPCR structure analysis platform to investigate the GPCR activation mechanisms. The authors analyzed all available 488 structures from different GPCR classes and presented a molecular map for GPCR activation, ligand binding, and G protein coupling.
The newly developed online GPCR structure analysis platform is useful and easy to navigate. Using this platform, the authors provided macro and micro switches for GPCR activation. The results suggested new activation switches along with activation switches that have been suggested by other studies. Overall, the manuscript suggests novel findings that would provide valuable information for the structural mechanism of GPCR activation. There are, however, a few issues that should be addressed to improve the manuscript.

Major comments
1. Although they have provided the detailed information as supplementary data, It would be nice if the authors provide more detailed information about activation structures (agonist-bound or G protein-bound or arrestin-bound states) analyzed in the manuscript text.

2. Would the analysis results provide different macro or micro switches that discern the G protein binding and arrestin binding or selectivity for G protein subtypes?

3. It has been suggested that there are sequential conformational changes during GPCR activation and G protein coupling, and the complex structures are final stage structures (DOI: 10.1016/j.cell.2019.04.022, 10.1016/j.cell.2019.04.021). Please consider discussing this issue along

with the analysis results of the manuscript.

4. Please provide experimental evidence for the newly suggested GPCR activation switches.

Minor comments
1. On page 4 and Fig. 3a class F, should it be 52% and 48%, not 62% (line 25) and 38%?
2. Please consider rewriting a sentence on page 5 line 15, “We next ... pairs confirms.”

Reviewer #2:
Remarks to the Author:
This manuscript by Kooistra et al., Babu and Gloriam provides an integrated and comprehensive analysis of the movements and transduction pathways shared and different among the four major GPCR families as they transit from inactivated to activated states. A great strength of the work is their use of distance pairs, rather than more traditional superposition of structures and RMSDs, to find conserved interactions and switches among inactive- and active-stabilizing residue pairs, and switch residues implicated in both. A key contribution will be the reduction to groups of these residues that define the states among each of the GPCR families. From these studies both granular analyses of transduction pathways emerge, as do more general features (e.g., that residue rotomer switches are rare, the centrality of TM Helix 3, the ubiquitousness of helix rotation, often over rotomer changes, and the greater similarities of among receptors within families than within active vs inactive states). Many of these will provide guidance to the community to drive specific research questions (e.g., what state is my receptor in, how to I drive that/interrupt that by mutation, what is such-and-so ligand doing…?). I thus find this a strong manuscript that will interest the community, and I support publication. As I have seen it previously (and liked it then), and since most of my key critiques have been addressed, I think the manuscript can be published as is.

Author Rebuttal to Initial comments

We thank all Reviewers for the insightful and positive comments which have improved the manuscript and online resources. We respond to each comment below. In addition, we have also incorporated recent structural templates for classes C and F (class A and B1 already had strong coverage) and updated all associated figures and text accordingly. We look forward to your opinion on the revised manuscript.

Reviewer #1:

Hauser et al. used a newly developed online GPCR structure analysis platform to investigate the GPCR activation mechanisms. The authors analyzed all available 488 structures from different GPCR classes and presented a molecular map for GPCR activation, ligand binding, and G protein coupling.

The newly developed online GPCR structure analysis platform is useful and easy to navigate. Using this platform, the authors provided macro and micro switches for GPCR activation. The results suggested new activation switches along with activation switches that have been suggested by other studies. Overall, the manuscript suggests novel findings that would provide valuable information for the structural mechanism of GPCR activation. There are, however, a few issues that should be addressed to improve the manuscript.

We thank the reviewer for the nice summary of the paper and its scope providing both macro and micro switches for GPCR activation, including many new activation switches (not reported for classes B1, C and F before). Furthermore, we highly appreciate the confirmation of that the novel findings provide valuable information about the structural mechanism of GPCR activation, and of the utility of the online structure analysis platform.

Major comments

1. Although they have provided the detailed information as supplementary data, It would be nice if the authors provide more detailed information about activation structures (agonist-bound or G protein-bound or arrestin-bound states) analyzed in the manuscript text.

We have added more detailed information about active structures in all places where specific receptors are discussed or where the type of complex could be expected to affect the conclusions. As it is not possible to list all complexed proteins and ligands for all active state receptors investigated, for the other places of the text we have added additional references to the relevant supplementary data. All representative active state structures used for the analyses are in the G protein-bound active state. No arrestin-bound states have gone into the analysis of the activation state comparisons.

2. Would the analysis results provide different macro or micro switches that discern the G protein binding and arrestin binding or selectivity for G protein subtypes?

This particular paper focuses on the common activation mechanisms that govern the stabilization of an inactive and active state, respectively. It solely uses G protein-bound templates, as these are at the top of the signaling cascade and have a shared structural scaffold allowing comparable active receptor

states, which is a prerequisite of our comparative structure analysis approach. Other studies have described molecular mechanisms (DOI: 10.1038/s41586-018-0077-3, 10.1038/s41594-018-0071-3) and motifs (DOI: 10.1038/s41467-019-09204-y, DOI: 10.1016/j.cell.2017.07.002) involved in GPCR-mediated arrestin activation.

It would indeed be intriguing to investigate G protein family specific determinants of binding and activation. However, there are not sufficient templates yet to do that in a comprehensive comparative fashion across the GPCR classes and G protein families. Such an analysis would, conversely to this, not identify common activation determinants for a given GPCR class but those unique to a G protein family. Such an analysis would therefore have a larger focus on determinants of G protein binding selectivity, which has also been studied previously (e.g. DOI: 10.1038/nature22070) using a sequence-based approach. We suggest that structure-based comparisons across GPCR classes and G protein families are revisited when the coverage by structural templates is improved.

3. It has been suggested that there are sequential conformational changes during GPCR activation and G protein coupling, and the complex structures are final stage structures (DOI: 10.1016/j.cell.2019.04.022, 10.1016/j.cell.2019.04.021). Please consider discussing this issue along with the analysis results of the manuscript.

Indeed, during GPCR activation more transient intermediate states can form and the complex structures in our analysis represent the stable nucleotide-free GPCR-G protein "final stage" complexes. To reflect this, we have added the following text to the Discussion:

It has furthermore been suggested that there are sequential conformational changes during GPCR activation and G protein coupling with transient intermediate states facilitating the transition of the extensive conformational rearrangement, which is not captured by currently available complex structures. (10.1016/j.cell.2019.04.022, 10.1016/j.cell.2019.04.021). The proposed intermediate state complexes may require additional state determinants beyond the ones identified herein. Hence, going forward, it will be important to combine structural studies with biophysical investigations such as FRET-based systems (https://doi.org/10.1038/nature22354), DEER- (10.1073/pnas.2013904117), NMR (10.1073/pnas.2009786117) or even mass spectroscopy (10.1016/j.sbi.2021.03.014) for monitoring specific interactions in more infrequent conformations.

4. Please provide experimental evidence for the newly suggested GPCR activation switches.

We have added mutagenesis data generated by Franziska M. Heydenreich, Dmitry B. Veprintsev and Michel Bouvier. This confirms that mutations of predicted state-changing residues have a larger effect on potency (but not on efficacy) than do predicted non-state changing residues. We also provide a structural mapping of the tested mutations showing a clustering in the transduction pathway bridging the ligand and G protein sites.

Minor comments

1. On page 4 and Fig. 3a class F, should it be 52% and 48%, not 62% (line 25) and 38%?

We thank the Reviewer for spotting this error. It has now been corrected.

2. Please consider rewriting a sentence on page 5 line 15, “We next ... pairs confirms.”

This sentence has been corrected to “*We next investigated single helix rearrangements across 13 receptor inactive/active state structure pairs.*”.

Reviewer #2:

Remarks to the Author:

This manuscript by Kooistra et al., Babu and Gloriam provides an integrated and comprehensive analysis of the movements and transduction pathways shared and different among the four major GPCR families as they transit from inactivated to activated states. A great strength of the work is their use of distance pairs, rather than more traditional superposition of structures and RMSDs, to find conserved interactions and switches among inactive- and active-stabilizing residue pairs, and switch residues implicated in both. A key contribution will be the reduction to groups of these residues that define the states among each of the GPCR families. From these studies both granular analyses of transduction pathways emerge, as do more general features (e.g., that residue rotomer switches are rare, the centrality of TM Helix 3, the ubiquitousness of helix rotation, often over rotomer changes, and the

greater similarities of among receptors within families than within active vs inactive states). Many of these will provide guidance to the community to drive specific research questions (e.g., what state is my receptor in, how to I drive that/interrupt that by mutation, what is such-and-so ligand doing…?). I thus find this a strong manuscript that will interest the community, and I support publication. As I have seen it previously (and liked it then), and since most of my key critiques have been addressed, I think the manuscript can be published as is.

We thank Reviewer 2 for reviewing this manuscript again, which has indeed been revised after review by another journal. We are very pleased to get the solid confirmation of the value of the new approaches, and that the analysis will be able to guide future studies seeking to answer yet unsolved questions about receptor activation.

Decision Letter, first revision:

12th Aug 2021

Dear David,

Thank you for submitting your revised manuscript "GPCR activation mechanisms across classes and macro/microscales" (NSMB-A44783A). It has now been seen by one of the original referees and their comments are below. The reviewer finds that the paper has improved in revision, and therefore we'll be happy in principle to publish it in Nature Structural & Molecular Biology, pending minor revisions to comply with our editorial and formatting guidelines.

We are now performing detailed checks on your paper and will send you a checklist detailing our editorial and formatting requirements in about a week. Please do not upload the final materials and make any revisions until you receive this additional information from us.

To facilitate our work at this stage, we would appreciate if you could send us the main text as a word file. Please make sure to copy the NSMB account (cc'ed above).

Thank you again for your interest in Nature Structural & Molecular Biology Please do not hesitate to contact me if you have any questions.

Kind regards,
Florian

Florian Ullrich, Ph.D.
Associate Editor
Nature Structural & Molecular Biology
ORCID 0000-0002-1153-2040

Reviewer #1 (Remarks to the Author):

The authors resolved all the concerns that I raised.

Final Decision Letter:

22nd Sep 2021

Dear David,

We are now happy to accept your revised paper "GPCR activation mechanisms across classes and macro/microscales" for publication as a Article in Nature Structural & Molecular Biology.

Acceptance is conditional on the manuscript's not being published elsewhere and on there being no announcement of this work to the newspapers, magazines, radio or television until the publication date in Nature Structural & Molecular Biology.

Before the manuscript is sent to the printers, we shall make any detailed changes in the text that may be necessary either to make it conform with house style or to make it intelligible to a wider readership. If the changes are extensive, we will ask for your approval before the manuscript is laid out for production. Once your manuscript is typeset you will receive a link to your electronic proof via email within 20 working days, with a request to make any corrections within 48 hours. Please read proofs with great care to make sure that the sense has not been altered. If you have queries at any point during the production process then please contact the production team at rjsproduction@springernature.com. Once your paper has been scheduled for online publication, the Nature press office will be in touch to confirm the details.

Please note that due to tight production schedules, proofs should be returned as quickly as possible to avoid delaying publication. If you anticipate any limitations to your availability over the next 2-4 weeks (such as vacation or traveling to conferences, etc.), please e-mail rjsproduction@springernature.com as soon as possible. Please provide specific dates that you will be unavailable and provide detailed contact information for an alternate corresponding author if necessary.

To assist our authors in disseminating their research to the broader community, our SharedIt initiative provides all co-authors with the ability to generate a unique shareable link that will allow anyone (with or without a subscription) to read the published article. Recipients of the link with a subscription will also be able to download and print the PDF.

As soon as your article is published, you can generate your shareable link by entering the DOI of your article here: <a href="http://authors.springernature.com/share">http://authors.springernature.com/share<a>. Corresponding authors will also receive an automated email with the shareable link

Note the policy of the journal on data deposition: http://www.nature.com/authors/policies/availability.html.

Your paper will be published online soon after we receive proof corrections and will appear in print in the next available issue. You can find out your date of online publication by contacting the production team shortly after sending your proof corrections. Content is published online weekly on Mondays and Thursdays, and the embargo is set at 16:00 London time (GMT)/11:00 am US Eastern time (EST) on the day of publication. Now is the time to inform your Public Relations or Press Office about your paper, as they might be interested in promoting its publication. This will allow them time to prepare an accurate and satisfactory press release. Include your manuscript tracking number (NSMB-A44783B) and our journal name, which they will need when they contact our press office.

About one week before your paper is published online, we shall be distributing a press release to news organizations worldwide, which may very well include details of your work. We are happy for your institution or funding agency to prepare its own press release, but it must mention the embargo date and Nature Structural & Molecular Biology. If you or your Press Office have any enquiries in the meantime, please contact press@nature.com.

You can now use a single sign-on for all your accounts, view the status of all your manuscript submissions and reviews, access usage statistics for your published articles and download a record of your refereeing activity for the Nature journals.

If you have not already done so, we strongly recommend that you upload the step-by-step protocols used in this manuscript to the Protocol Exchange. Protocol Exchange is an open online resource that allows researchers to share their detailed experimental know-how. All uploaded protocols are made freely available, assigned DOIs for ease of citation and fully searchable through nature.com. Protocols can be linked to any publications in which they are used and will be linked to from your article. You can also establish a dedicated page to collect all your lab Protocols. By uploading your Protocols to Protocol Exchange, you are enabling researchers to more readily reproduce or adapt the methodology you use, as well as increasing the visibility of your protocols and papers. Upload your Protocols at www.nature.com/protocolexchange/. Further information can be found at www.nature.com/protocolexchange/about.

An online order form for reprints of your paper is available at https://www.nature.com/reprints/author-reprints.html. Please let your coauthors and your institutions' public affairs office know that they are also welcome to order reprints by this method.

Please note that <i>Nature Structural & Molecular Biology</i> is a Transformative Journal (TJ). Authors may publish their research with us through the traditional subscription access route or make their paper immediately open access through payment of an article-processing charge (APC). Authors will not be required to make a final decision about access to their article until it has been accepted. Find out more about Transformative Journals

<B>Authors may need to take specific actions to achieve compliance with funder and institutional open access mandates. For submissions from January 2021, if your research is supported by a funder that requires immediate open access (e.g.

according to Plan S principles) then you should select the gold OA route, and we will direct you to the compliant route where possible. For authors selecting the subscription publication route our standard licensing terms will need to be accepted, including our self-archiving policies. Those standard licensing terms will supersede any other terms that the author or any third party may assert apply to any version of the manuscript.

In approximately 10 business days you will receive an email with a link to choose the appropriate publishing options for your paper and our Author Services team will be in touch regarding any additional information that may be required.

You will not receive your proofs until the publishing agreement has been received through our system.

If you have any questions about our publishing options, costs, Open Access requirements, or our legal forms, please contact ASJournals@springernature.com

Kind regards,
Florian

Florian Ullrich, Ph.D.
Associate Editor
Nature Structural & Molecular Biology
ORCID 0000-0002-1153-2040

Click here if you would like to recommend Nature Structural & Molecular Biology to your librarian:
http://www.nature.com/subscriptions/recommend.html#forms
